# Assessing the integration of social information using AI-generated videos: a brief research report

**DOI:** 10.3389/fpsyg.2025.1505843

**Published:** 2026-02-06

**Authors:** Paul Compensis

**Affiliations:** Institute of Psychology, University of Bamberg, Bamberg, Germany

**Keywords:** AI-generated stimuli, facial emotion expressions, social categories, social cues, social expectation violations

## Abstract

Integrating social information from different cues and modalities is a central feature of social functioning and humans respond sensitively to incongruences between different social information types (e.g., smiling while uttering a negative statement about oneself). To investigate the processing of these integration processes, researchers frequently draw on the simultaneous or consecutive presentation of static stimuli, such as written text and images, which often exhibit a relatively low naturalness of these stimuli. In this brief research report, I illustrate how AI-generated videos can successfully be used to overcome some of these limitations. Videos were generated by combining different avatar images (that were also generated with an AI tool) with text messages that either presented statements that were congruent or incongruent with expectations based on the age, gender, or facial emotion expressions of the avatar. In this pilot study, 47 participants watched these videos and rated the plausibility and emotional intensity of each utterance. Despite being fully generated artificially, videos elicited different responses depending on the condition. Incongruences based on social categories (age, gender) engendered lower plausibility ratings while intensity ratings were relatively unaffected. In contrast, intensity ratings for emotion expressions were expectedly higher (irrespective of congruence) but sensitivity to plausibility was affected by the congruence of the facial expression and the statement of the avatar. This pilot study illustrates the potential of AI-generated stimuli for investigating socio-cognitive processes, particularly in research on the integration of social information.

## 1 Introduction

Humans typically strive for at least a certain level of consistency in the behavior of others (Fiske and Taylor, 2021; Heider, 1958) and react sensitively to incongruent information in social interaction (Bartholow et al., 2001; Berman et al., 1983). Incongruent information is occasionally encountered when two different social information signals do not match, often eliciting a particular response in the perceiver. For instance, observing someone grinning while watching negative or disturbing imagery makes us rather feel odd (Szczurek et al., 2012; see also Kastendieck et al., 2021; Olivera-La Rosa et al., 2021). Likewise, when someone utters a sentence such as “Today, she left on the trip of her dreams” while making a sad or fearful face most likely leaves a weird impression (provided there is no additional context that could explain the unexpected behavior) and elicits particular cognitive responses in the perceiver (Dozolme et al., 2015; see also Zaki et al., 2010). Similar effects are found for incongruences between social categories such as age, gender, or social class and reported behavior of a person, for instance, when a 6-year-old girl utters a sentence such as “Every evening, I drink a glass of wine before I go to sleep” (van den Brink et al., 2012).

As can be seen in these examples, humans rely on a wide number of social information types to evaluate the behavior of others and draw inferences about their intentions. In the domain of non-verbal social cues, eye gaze is an important index for the current attention of people we interact with (Langton et al., 2000) while facial emotion expressions as well as voice carry important information about the emotional states of another person (McKone and Robbins, 2011; Schirmer and Adolphs, 2017). Humans also draw on social category information such as age, (perceived) sex, and group membership (Brooks and Freeman, 2024) to draw conclusions about others. These social categories can be derived from different sources, such as the face (e.g., Rule and Sutherland, 2017), clothing style (Reid et al., 1997), or voice (see the overview in Guyer et al., 2021). Ultimately, humans also take into account the behavior displayed by others (Teoh et al., 2017) and this behavior can also be manifested in what people reveal about themselves. Telling colleagues about the crazy party last weekend is as much telling as emphasizing my participation in a marathon.

It is a central aspect of social functioning to perceive and integrate social information from different sources into a coherent picture. The examples above contained incongruences that impair the successful integration of incoming information, disallowing accurate impression building and obstructing the drawing of inferences about another person. Processing such inconsistencies is associated with particular neuro-cognitive responses (see, for instance, Ma et al., 2012; Mende-Siedlecki and Todorov, 2016) and engenders elaborate (domain-general and domain-specific) conflict resolution processes (e.g., Zaki et al., 2010).

Using this form of incongruence or violation paradigms to study integration processes in social cognition proved to be a fruitful experimental procedure (Brannon and Gawronski, 2018). A major drawback of many previous studies, however, is the reliance on static visual and textual stimuli. In some cases, these stimuli are even presented separately, either consecutively (e.g., sentence presentation followed by an image, as in Dozolme et al., 2015) or one above the other (e.g., Zaki et al., 2010), clearly reducing the naturalness and ecological validity of these studies. Other studies achieve a more natural presentation by resampling videos elicited in response to one stimulus type (e.g., positive images) with stimuli of the opposite valence (e.g., negative images) to produce emotional incongruence (e.g., Szczurek et al., 2012). While this procedure results in more natural material, its production is relatively costly and still does not fully capture the presentation of social signals that, in real life, appear in a dynamic and synchronous way. In addition, producing stimuli with human actors does not allow for full control over all parameters.

Lucky, these three limitations (staticity, costs, control) become more and more obsolete due to the advent of recent forms of artificial intelligence (AI) and particularly large language models (LLMs), with GPT-5 (OpenAI, 2025) and Gemini (Google, 2023) being currently the most widely known models. LLMs can be used to generate large numbers of different stimuli types in a very fast and cost-efficient way (e.g., Peres et al., 2023). While AI-based chatbots (such as ChatGPT) can generate a large number of text stimuli of a particular form (e.g., text stimuli of a particular word length), LLMs modified for text-to-image generation (e.g., DALL-E 3 by OpenAI, 2023a) can produce realistic images of people of a specified category (e.g., being young), showing a particular emotional expression (e.g., smiling), or performing a specific task (e.g., waving). Even more powerful in this regard are more recently emerging text-to-video models (e.g., Kling by Kuaishou, 2024). Particularly valuable for research into social interaction and social cognition are tools that allow for the integration of written text and images into a dynamic video sequence, and this is the main purpose of the present contribution.

In this brief report, I want to demonstrate how AI-generated stimulus material—and in particular AI-generated videos—can be applied to the assessment of socio-cognitive information integration processes. To illustrate the use of AI-generated video stimuli, I draw on the aforementioned research on integration processes of social information that typically combined a social cue with a statement about or utterance of the target person. For instance, Dozolme et al. (2015) first presented a descriptive sentence (“Today, she left on the trip of her dreams”) and then a congruent (happy) or incongruent (sad) facial expression. In their study, incongruent stimuli decreased accuracy and speed on a subsequent evaluation task (and engendered a mismatch-related N400 event-related potential component), an effect that was also modulated by trait empathy. Similarly, Zaki et al. (2010) presented negative or positive statements (“My dog died.”) alongside a congruent or incongruent facial emotion expression while subjects were undergoing functional magnetic resonance imaging (fMRI). Here, different social cognitive conflict resolution mechanisms were assessed by comparing the combined presentation of image and utterance to the presentation of either text or face alone.

As was mentioned above, not only emotion cues but also social categories are ideal testing candidates to assess integration processes. Van den Brink et al. (2012) tested whether age, gender, and social class (inferred from the voice of a speaker) affect the processing of an utterance that either matches or mismatches expectations concerning the social category (for instance, a child’s voice saying that she is drinking a glass of wine before going to sleep). An additional interesting aspect of the study by van den Brink et al. (2012) is that they compared social-based incongruencies to purely meaning-based lexical-semantic violations (such as “You wash your hands with horse and water.”), allowing for the discrimination of linguistic violations from social information-based conflict processing. Another example is Portengen et al. (2024), who presented parents with pictures of their own or unknown children followed by words (“race car,” “princess dress”) that are considered congruent or incongruent with gender-stereotypes (with gender-stereotype violations of their children eliciting a stronger behavioral and neurophysiological response).

In the present contribution, I present the results from pilot work that used AI-generated videos of avatars telling something about themselves. Videos were generated by combining avatar images (that were likewise generated by AI) with messages that either presented a neutral statement or a statement that was congruent or incongruent with expectations based on the age, gender, or facial emotion expressions of the avatar. In each trial, participants rated the video with respect to plausibility and emotional intensity (but no psychophysiological measure was taken here). Based on the assumption that AI-generated videos are highly valid and immersive stimuli (Becker and Laycock, 2023), I hypothesize that videos with incongruent information were rated as less plausible than congruent trials, with social category incongruences receiving a lower plausibility rating than emotional expression incongruence. For emotional intensity, I expect a higher rating for emotional expression conditions compared to social category trials, irrespective of the congruence of cue and utterance.

## 2 Methods

### 2.1 Materials

For this pilot study, brief videos with avatars uttering one sentence were created using AI-based video tools by D-ID (based on GPT-3.5-turbo, OpenAI, 2023b).^1^ In a first step, the avatar generation tool implemented into D-ID’s video studio was used to create images of avatars depicting people of different age and sex (e.g., see Table 1). To this end, prompts such as “a 7-year old girl with red hair, facing the viewer” or “a 39-year old man with a happy face” were used to generate a number of avatar images.

**Table 1:** Example material for each condition.

Dimension	Social category (SC)	Facial emotion expression (FE)	Lexical-semantic content (LS)
Example frame from the videos	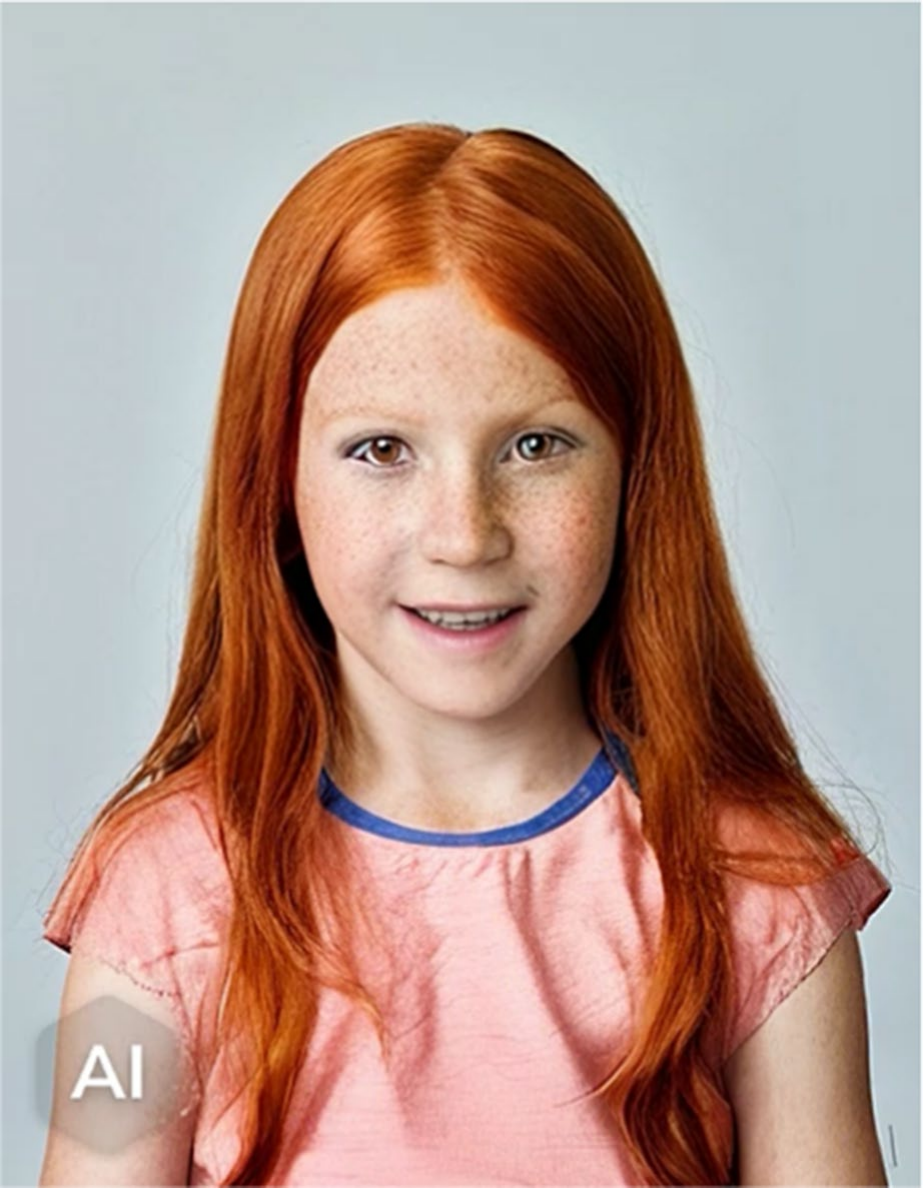	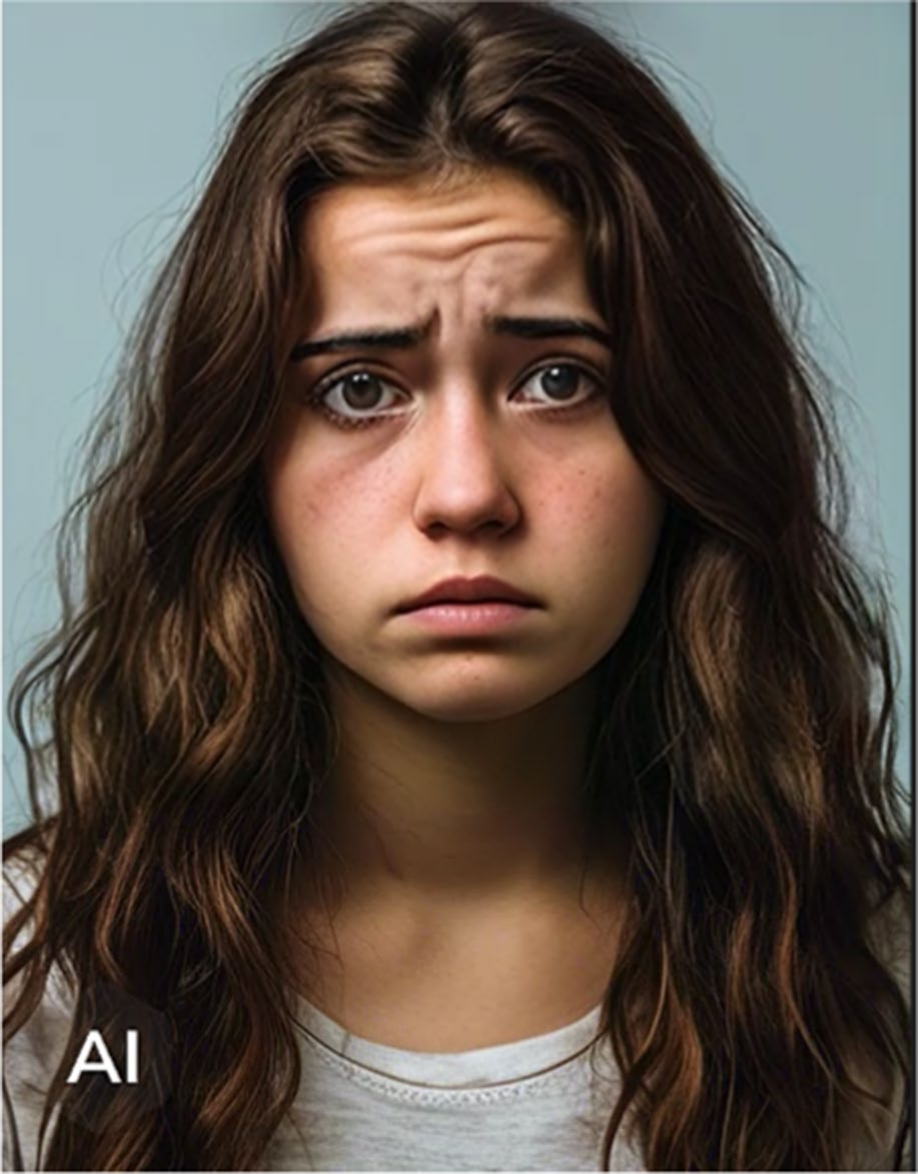	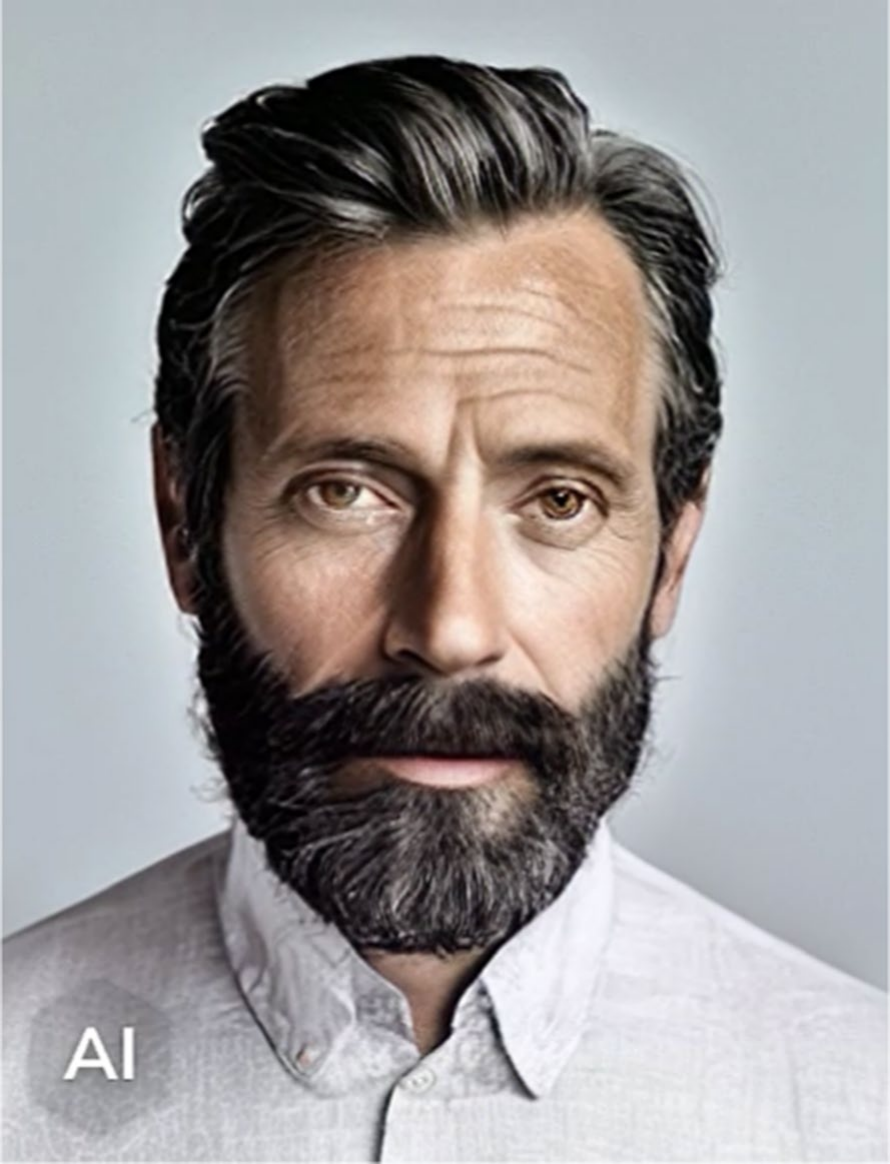
Congruent	*Jeden Abend trinke ich ein Glas Milch.* *I drink a glass of milk every evening.*	*Ich habe eine schlechte Note bekommen.* *I received a bad grade.*	*Ich gehe gerne Angeln.* *I like to go fishing.*
Incongruent	*Jeden Abend trinke ich ein Glas Wein.* *I drink a glass of wine every evening.*	*Ich habe die Klausur bestanden.* *I passed the exam.*	*Morgens esse ich Sägespähne.* *I eat sawdust in the morning.*

It is important to note here that AI-generated videos may reflect biases in gender, age, and ethnicity, as has been observed in textual data (e.g., Ayoub et al., 2024; Fang et al., 2024). In this pilot study, only age and sex were systematically controlled, and ethnic representation was not specified. While this limitation is unlikely to have a major impact in the context of our German psychology student sample, it highlights the need for careful consideration of potential biases in future work.

In a few cases, this generation tool produced unnaturally looking avatars. These images were discarded and the prompts repeated or slightly alternated to generate more adequate images (note that as with other AI tools, the image generation tool implemented in D-ID does not allow for the generation of exactly the same images twice). In a second step, I selected artificial German voices from the voice data bases integrated into D-ID’s video generation tool (that are taken from larger voice data bases such as Microsoft’s Azure Voices).^2^ In total, I prepared 48 images for the purpose of this pilot study (with avatars’ sex distributed equally and age as well as emotion expression alternated as required for the relevant study conditions).

In a next step, I manually prepared text stimuli in the form of utterances of five to seven words. Each utterance was a brief statement describing a person by themselves in the first person (for instance, “I love my dog” or “I passed the exam”). These utterances were then added to the script field of the AI tool and videos were created by using the video-from-image function of D-ID’s video studio. Each resulting video was checked and assessed for consistency manually. Visually checking is particularly relevant because the generation algorithm adds subtle body movement and blinking to the avatars (calculated from the images), in some cases leading to miscalculations (that is, impossible lip or implausible eye movements). In such cases, I repeated the procedure with the same text and image until a more adequate video was produced by the algorithm (again, repeating the procedure will not reproduce exactly the same result twice).

Following the procedure described so far, 48 pilot trials were created. The pilot study had a 3 × 2 design with the two factors dimension of evaluation (social category, SC; facial emotion expression, FE; lexical-semantic content, LS) and congruence (match vs. mismatch). Congruence or incongruence in each dimension evolved from the interplay of the person shown in a video and the utterance spoken by that person (example trials for each condition are provided in Table 1). Sixteen trials were associated with social identity integration. Avatars in this condition had a neutral expression. Eight of them depicted relatively young (below 12) or relatively old people (above 65) who either uttered a statement that is in line with age-based social expectations (e.g., a young girl saying “I drink a glass of milk every evening”) or violates these expectations (the same girl saying “I drink a glass of wine every evening”; see also the second column in Table 1).

Eight additional avatars represented middle-aged (20–50 years old) people with a neutral face who uttered sentences congruent or incongruent with gender-based expectations (e.g., a woman vs. a man saying “Tomorrow I have to see my gynecologist.”). The following 16 trials were associated with emotion expressions. Eight avatars made a happy face (and four each uttered a sentence that is either congruent or incongruent with the emotion expressed; for examples see the third column in Table 1). The last 16 trials consisted of avatars with a neutral expression (again with gender balanced) uttering either sentences with simple lexical-semantic violations (“Tomorrow I fry sand.”) or perfectly grammatical and adequate sentences (“I love my dog.”).

For the purpose of a brief exploratory analyses, participants also conducted the Reading the Mind in the Eyes task (RMET) (Baron-Cohen et al., 2001), a task that is traditionally understood as a measure of intention-reading skills and cognitive empathy. In the RMET, participants see black-and-white images of only the eye region of individuals. They then select one out of four adjectives or descriptives that most adequately describe the intentional or emotional states presented by the eyes.

### 2.2 Procedure

Data collection took place as part of a larger study assessing several measures of socio-cognitive functioning such as the RMET and the Yoni task (Shamay-Tsoory and Aharon-Peretz, 2007) but also narrative text elicitations for the assessment of implicit processes (see Feffer et al., 2008) but these measures are not discussed in the present contribution.

A total of 47 participants (2 identifying as male) with a mean age of 21.17 years (*SD* = 4.09) were recruited at the University of Erlangen-Nuremberg. The study relied on a convenience sample, as it constituted pilot work in preparation for a larger follow-up project. Participants were primarily students, resulting in a relatively high overall education level. Given the pilot nature of the study, no prior power analysis was conducted, and due to the predominance of female participants, no *post-hoc* analyses by sex or gender were performed. Clearly, considering potential interactions between a participant’s sex or gender and the (perceived) sex of the avatar or other stimulus material (e.g., Fischer et al., 2004), future studies should aim for a more gender-balanced distribution in the sample.

After providing informed consent, participants were seated in front of a computer in single lab cabins. They first worked on the other tasks of the larger study for roughly about an hour. Then, the pilot task started. Stimuli were presented with a presentation script built and run with Inquisit 6.0.^3^ Videos were presented in the center of a white screen. Each participant saw each of the 48 different trials in randomized order. Participants were instructed to first simply watch the video while the avatar of the videos makes their brief statement. Then, participants were asked to rate the overall plausibility as well as the emotional intensity of the utterance of the person in the video. For this purpose, slider elements with ranges from “very plausible”/“highly intense” on the right side to “not at all plausible”/“not intense” on “the left side” were presented. The range of both sliders ranged from zero to 100 but the number values of this scale were not visible to the participants. The main reason to use a slider with a broad interval was to allow for more fine-grained ratings for each item. Each trial was presented in this manner and participants were then redirected to the next (and final) part of the overall study (asking for demographic data).

All data pre-processing and analysis was conducted in *R* (R Core Team, 2023). Means and standard deviations of the plausibility and intensity ratings were calculated for each condition. Note that neither plausibility ratings (*W* = 0.86, *p* < 0.001) nor intensity ratings (*W* = 0.95, *p* < 0.001) were normally distributed in this sample, as determined by Shapiro–Wilk tests. However, visual inspection of Q-Q plots and residuals-versus-fitted values suggested approximate normality of residuals. Given the robustness of linear mixed-effects models to minor deviations from normality, these models were used for hypothesis testing. For these calculations, the *lmer()* function from the *R* packages “*lme4” was used* (Bates et al., 2015). The models included the fixed factors DIMENSION and CONGRUENCE and random effects for subject and item to account for idiosyncratic or item-specific use of the rating scales (but no random slopes were specified for either model). *Post-hoc* analyses for both models were subsequently conducted using the *emmeans()* function from the *R* package of the same name (Lenth, 2023), with the specification to contrast congruent versus incongruent trials. Pairwise comparisons were performed with a Tukey adjustment for multiple comparisons. Estimated marginal means are plotted for visualization using the *ggplot2()* package (Wickham, 2016).

For exploratory purposes, Pearson correlations were calculated for plausibility and intensity ratings in each condition with individual RMET scores. In the present sample, the RMET was normally distributed (*W* = 0.99, *p* = 0.48) and showed acceptable internal consistency (Cronbach’s *α* = 0.71).

## 3 Results

In the plausibility ratings, means and standard deviations were relatively comparable across the three dimensions when the utterance matched expectations based on social category (age or gender), emotion expression (happiness or sadness) or lexical-semantic rules. In congruent contexts, plausibility ratings were the highest, on average, for the SC condition (*M* = 86.4, *SD* = 14.9), directly followed by LS (*M* = 85.2, *SD* = 17.4) and FE (*M* = 83.4, *SD* = 19.0). In contrast, ratings differed more strongly when there was incongruence within the different dimensions, but ratings also differed across dimensions. FE mismatches received the highest rating of the incongruent conditions (*M* = 43.5, *SD* = 29.3) followed by SC (*M* = 36.2, *SD* = 31.6). Lexical-semantic violations received the lowest ratings (*M* = 3.34, *SD* = 9.59).

**Figure 1:**
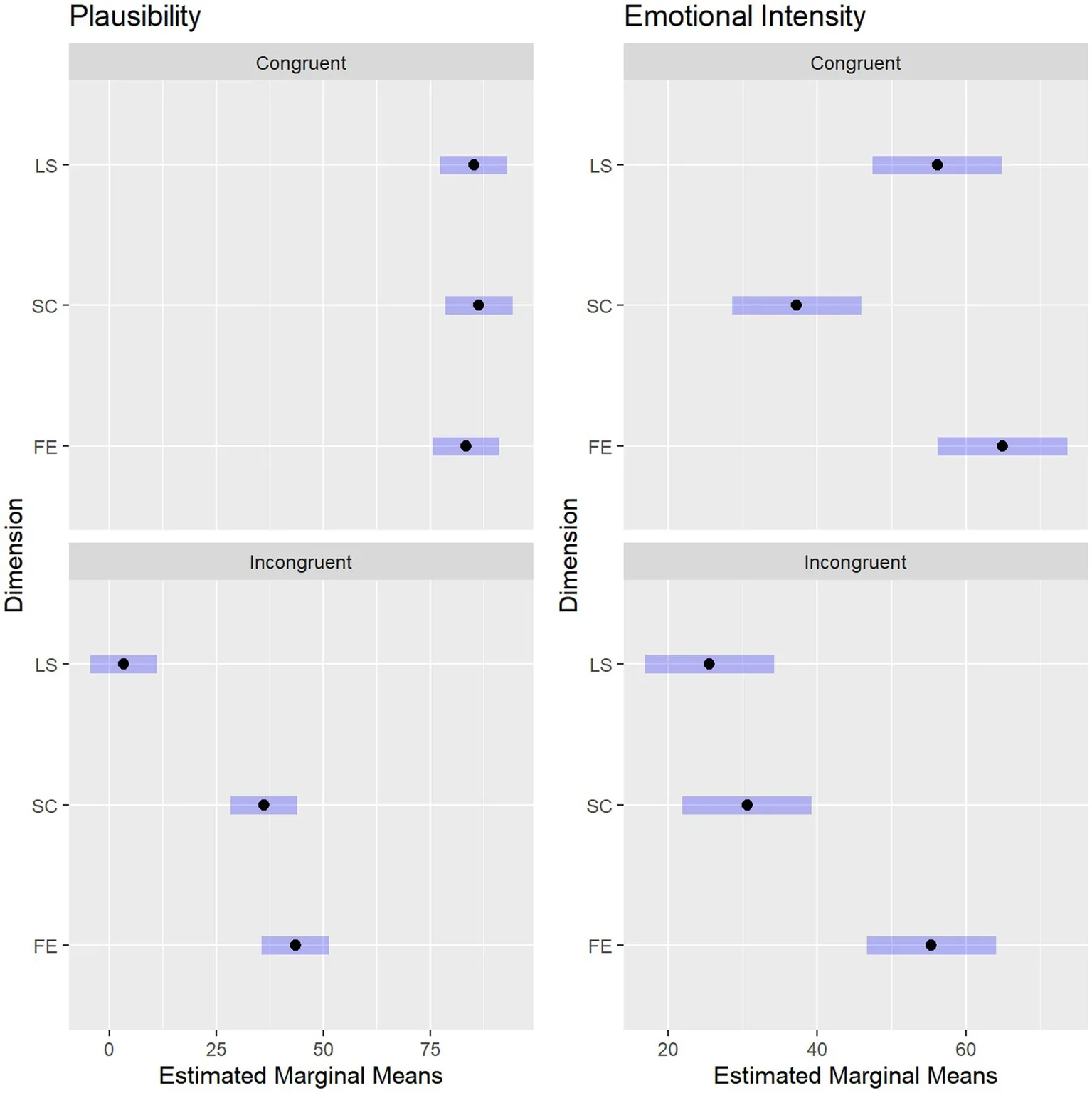
Visualization of estimated marginal means for both models.

Linear mixed-effects models revealed significant main effects of dimension [χ^2^(2) = 35.32, *p* < 0.001, η^2^_p_ = 0.42 (95% CI: 0.24, 1.00)] and congruence [χ^2^(1) = 395.05, *p* < 0.001, η^2^_p_ = 0.89 (95% CI: 0.84, 1.00)], and a significant interaction between these factors [χ^2^(2) = 38.22, *p* < 0.001, η^2^_p_ = 0.44 (95% CI: 0.26, 1.00)]. *Post-hoc* analyses showed that none of the dimension contrasts are significant in the case of congruence between cues. In the case of incongruence, however, the comparisons between LS with FEs and with SCs are highly significant in both cases, with estimated differences of 40.14 (*p* < 0.001*) and 32.82 (*p* < 0.001*). In contrast, the estimated marginal means (EMM) difference between FEs and SIs (7.32) was not significant (*p* = 0.361). EMMs for all models are plotted in Figure 1.

In contrast, emotional intensity ratings were more sensitive to the type of dimension. Emotion expressions that matched the content of the utterance received the highest ratings (*M* = 64.9, *SD* = 26.2) while FEs with incongruent utterances received an average rating of 55.4 (*SD* = 26.2). SC received rather low ratings in both versions (congruent: *M* = 37.3, *SD* = 23.5; incongruent: *M* = 25.6, *SD* = 23.9). In contrast, intensity ratings differed strongly by cue congruence in lexical-semantic contexts. Grammatically correct and neutral utterances received a medium rating of 56.2 (*SD* = 23.0) while lexical-semantic violations received the lowest ratings (*M* = 25.6, *SD* = 23.9).

There were again significant main effects of dimension type [χ^2^(2) = 50.89, *p* < 0.001, η^2^_p_ = 0.52 (95% CI: 0.34, 1.00)] and congruence [χ^2^(1) = 25.20, *p* < 0.001, η^2^_p_ = 0.35 (95% CI: 0.17, 1.00)], but the (yet significant) interaction of type and congruence was smaller on emotional intensity [χ^2^(2) = 11.79, *p* = 0.003, η^2^_p_ = 0.20 (95% CI: 0.04, 1.00)]. In *post-hoc* analyses using estimated marginal means, the difference between FEs and SIs is significant in both versions with a difference of 27.63 (*p* < 0.001***) when cues were congruent and 24.76 (*p* < 0.001***) when they were incongruent. In contrast, the difference between FEs and LS is only significant for mismatches (29.81, *p* < 0.001***) while the difference between SI and LS is only significant for congruence (−18.88, *p* < 0.005**). Again, these contrasts are visualized in Figure 1.

**Figure 2:**
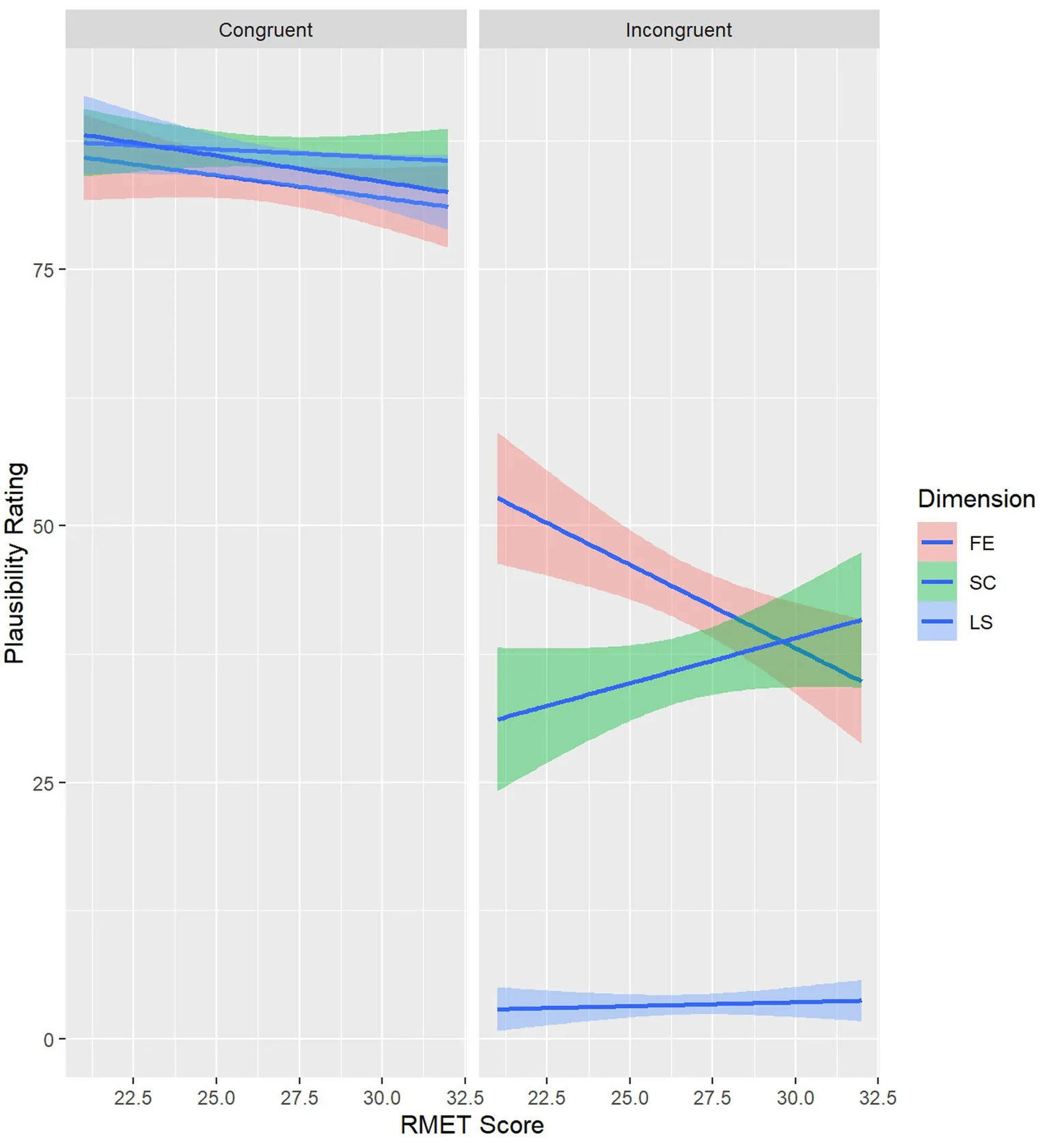
Exploratory assessment of potential influences of cognitive empathy.

For exploratory purposes, I calculated additional models for the variable plausibility rating by adding the participants’ scores on the RMET task (*M* = 26.68, *SD* = 2.93) as an additional factor to the factor dimension (but split by convergence) to assess whether individual differences in cognitive empathy interfere with the sensitivity to cue integration in the present task. There were no significant effects in the case of congruent cue—context matches. For incongruence, there was a significant main effect of dimension type [χ^2^(2) *=* 37.35, *p <* 0.001***] and a significant interaction of type and RMET score [χ^2^(2) *=* 26.78, *p <* 0.001***] but no main effect of RMET alone on plausibility. Nevertheless, upon closer examination split by dimension as well, effects of RMET remain only as a (non-significant) tendency, with higher RMET scores predicting slightly higher plausibility ratings for social identity incongruence [main effect of RMET score: χ^2^(1) *=* 1.65, *p* = 0.198] and lower ratings for emotion expression incongruence, χ^2^(1) *=* 2.50, *p* = 0.114, while lexical-semantic violations are not affected at all [χ^2^(1) *=* 0.08, *p* = 0.774]*. These tendencies are* visualized in Figure 2 (for the sake of simplicity, only as linear regression plots). Note that I also applied the same procedure to the other task variable intensity but here only the significant effect of dimension was present and no main or interaction effect of the RMET score (irrespective of congruence) was present.

## 4 Discussion

In this brief research report, I wanted to illustrate how AI-generated videos can successfully be applied to the investigation of socio-cognitive processes, in particular the integration of social information. To meet these ends, I elaborated on earlier paradigms brought forward by van den Brink et al. (2012) and Dozolme et al. (2015), and others. AI-generated video avatars of different age and gender and with different facial emotion expressions uttered sentences that were congruent or incongruent with the social category or emotional expression of the avatar. Different responses in the different conditions were assessed with plausibility and emotional intensity ratings.

In general, this procedure, despite being primarily pilot material, resulted in condition-specific differences in both ratings. Utterances that were in line with the social category or emotion expression or generally neutral sentences uttered in a neutral way were rated as highly plausible. As hypothesized, videos with incongruent information were generally rated as less plausible than congruent trials. More specifically, there was a gradient plausibility rating in trials with informational incongruence. As was predicted, social category incongruences were rated as (albeit non-significantly) less plausible than emotion incongruences. Interestingly, lexical-semantic violations, included in this study as a control condition, received the lowest ratings—suggesting that participants might be more sensitive to “language-internal” structural violations than to pragmatic ones, since the latter may often be reconciled through alternative interpretations such as irony or expressive intent.

The use of a second rating (emotional intensity) provided additional information leading to a more fine-grained pattern. For each information type, the presence of incongruence reduced intensity ratings to some extent but global differences in intensity across information type were larger. As was expected, emotional expression contexts were rated as more intense irrespective of congruence of cue and utterance. In contrast to emotional expressions, social category trials (with a neutral facial expression) were rated as rather less intense, likewise irrespective of congruence. It is also an interesting finding that lexical-semantic violations yielded the lowest intensity ratings while correct sentences (yet presented in a neutral way) were rated as comparably intense. This could suggest a potential link between linguistic-semantic violations and the processing of emotions but could also be explained by idiosyncratic differences in the respective stimuli material.

Taking together the two rating scales, this demonstrates that the different video stimuli are perceived differently depending on the condition. Lexical-semantic violations impact both plausibility and intensity ratings in a way that they are relatively high with matches and relatively low with mismatches. In contrast, incongruence with social identity and emotional information was subtler. While violations of social identity cues are reflected in the plausibility rating they receive only lower intensity ratings that are just slightly decreased by incongruence. In contrast, intensity ratings for FEs are expectedly high but are also sensitive to plausibility. This demonstrates the usability of AI-generated video stimuli for the assessment of social-cognitive information integration processes.

Since this study presented pilot work, there are naturally a number of limitations. Only a relatively small number of items was tested here. This is particularly problematic given the fact that I used a 2 × 3 design that even had some sub-components. For more robust testing, more trials are needed and, as was done in previous work, maybe the focus should be on only two of the conditions in comparison to each other to reduce unwanted cross-effects. In this study, gender and age were combined into a single dimension (social category), which may not be fully justified. Gender-based expectations (e.g., a woman saying “I am wearing a tie at work”) can often be more easily updated or perceived as plausible than age-based expectations (e.g., a six-year-old saying “I have a glass of wine”). Moreover, gender- and age-based stereotypes likely rely on different cognitive mechanisms and therefore combining them might complicate comparability (also since this resulted in uneven cell sizes in comparison to the other dimensions). Future studies should address this by clearly distinguishing between age- and gender-related factors.

Another limitation concerns the prior evaluation of the stimulus material, particularly regarding the (perceived) naturalness and congruency with respect to the relevant dimensions. This is especially important for socio-culturally sensitive aspects, such as gender and age, as well as for ensuring consistency in relation to the artificially generated material used in studies like the present one. Future studies could address this by incorporating prior ratings from independent reviewers or including a naturalness rating by participants as part of the study to ensure validity of the material.

In a similar vein, the size of the study sample was also not exceedingly high and the sample was relatively homogenous (high education and relatively high RMET scores) and predominantly female, restricting the transferability to other samples. As noted above, given potential interactions between a participant’s sex or gender and the stimulus material, future studies incorporating social categories should aim for a gender-balanced sample, even when gender is not the primary variable of interest.

Also, for future studies, the degree to which contextual information leads to consistent expectations must be controlled more consistently. In addition to ratings, more indirect tasks (such as self-paced reading to assess reaction time to single words) or psychophysiological measures should be used. Similarly, it is a non-trivial question how to assess interpersonal trait aspects, such as empathy or perspective-taking. In the present study, the classical RMET was used and demonstrated sufficient internal consistency. However, it should be noted that recent work has questioned the construct validity of the RMET, suggesting that it may primarily measure emotion recognition rather than more cognitively conceptualized aspects of perspective-taking or theory of mind (Oakley et al., 2016).

Despite limitations in the study procedure, this brief report clearly demonstrates that AI-generated videos can successfully be applied to research into social interaction and particularly to the area of integrating social information from different sources and modalities. AI tools provide a straightforward and cost-efficient way to generate dynamic stimulus material that can be more closely controlled than stimuli using human actors, while still preserving a substantial degree of ecological validity. The technical possibilities in this domain will certainly increase drastically in the coming years but the applicability of tools for research is already remarkable now. With the present contribution, I wanted to illustrate one of the many ways of using AI-generated stimuli for research on social interaction and communication.

## Data Availability

The raw data supporting the conclusions of this article will be made available by the author upon request, without undue reservation.

## Footnotes

1
https://www.d-id.com/

2
https://speech.microsoft.com/

3
https://www.millisecond.com
