# Correction: Assessing the integration of social information using AI-generated videos: a brief research report

**DOI:** 10.3389/fpsyg.2026.1827570

**Published:** 2026-10-19

**Authors:** Paul Compensis

**Affiliations:** Institute of Psychology, University of Bamberg, Bamberg, Germany

**Keywords:** AI-generated stimuli, facial emotion expressions, social categories, social cues, social expectation violations

There was a mistake in Table 1 as published. In the third column of the table, the two example sentences were swapped. The sentence “Ich gehe gerne Angeln” (“I like to go fishing”) is an example of a congruent sentence, whereas “Morgens esse ich Sägespäne” (“I eat sawdust in the mornings”) is an example of an incongruent sentence. The corrected Table 1 appears below.

**Table 1:** Example material for each condition.

Dimension	Social category (SC)	Facial emotion expression (FE)	Lexical-semantic content (LS)
Example frame from the videos	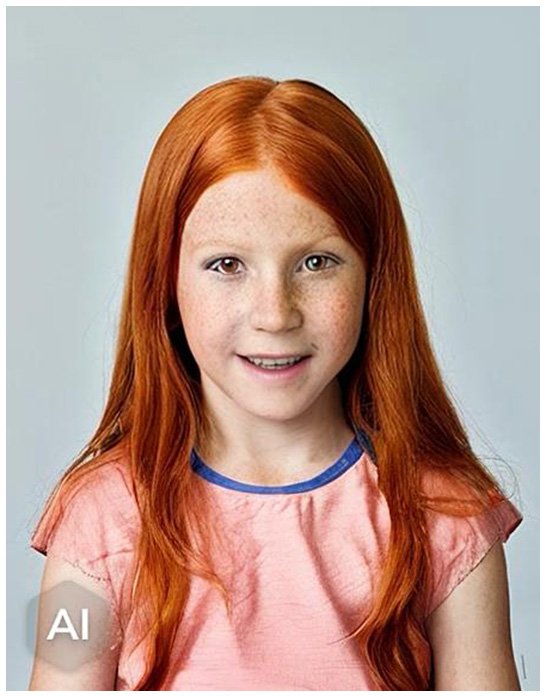	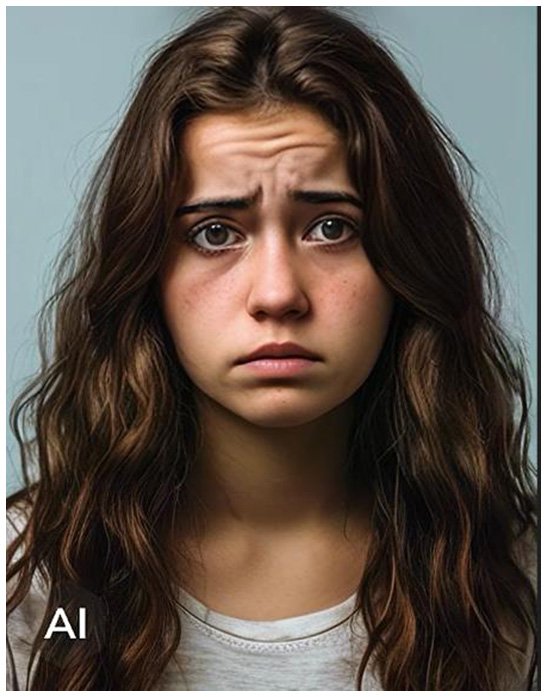	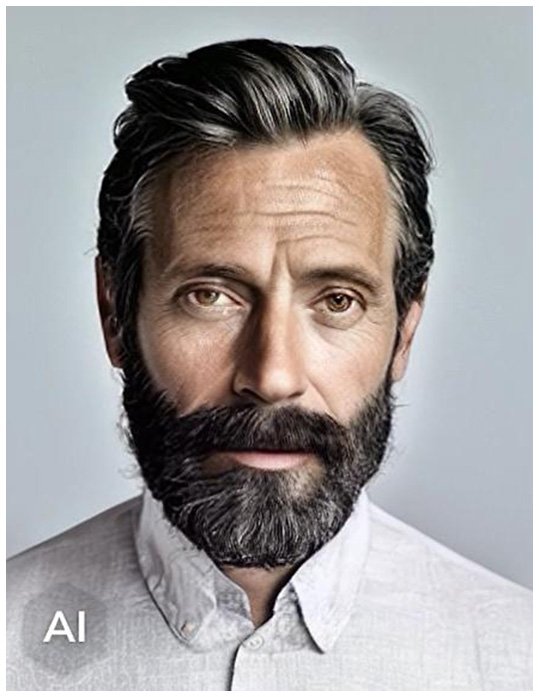
Congruent	*Jeden Abend trinke ich ein Glas Milch. I drink a glass of milk every evening*.	*Ich habe eine schlechte Note bekommen. I received a bad grade.*	*Ich gehe gerne Angeln. I like to go fishing*.
Incongruent	*Jeden Abend trinke ich ein Glas Wein. I drink a glass of wine every evening*.	*Ich habe die Klausur bestanden. I passed the exam*.	*Morgens esse ich Sägespähne. I eat sawdust in the morning*.

The original version of this article has been updated.

